# Protective Effect of *Potentilla glabra* in UVB-Induced Photoaging Process

**DOI:** 10.3390/molecules26175408

**Published:** 2021-09-06

**Authors:** Long You, Mi-Yeon Kim, Jae Youl Cho

**Affiliations:** 1 Department of Integrative Biotechnology, and Biomedical Institute for Convergence at SKKU (BICS), Sungkyunkwan University, Suwon 16419, Korea; youlonghc@gmail.com; 2 School of Systems Biomedical Science, Soongsil University, Seoul 06978, Korea; 3 Department of Biocosmetics, Sungkyunkwan University, Suwon 16419, Korea

**Keywords:** AP-1, moisturizing, photoaging, *Potentilla glabra* var. *mandshurica* (Maxim.) Hand.-Mazz., skin protection

## Abstract

Maintaining skin homeostasis is one of the most important factors for skin health. UVB-induced skin photoaging is a difficult problem that has negative impacts on skin homeostasis. So far, a number of compounds have been discovered that improve human skin barrier function and hydration, and are thought to be effective ways to protect skin homeostasis. *Potentilla glabra* var. *mandshurica* (Maxim.) Hand.-Mazz. Ethanol Extract (Pg-EE) is a compound that has noteworthy anti-inflammatory properties. However, its skin-protective effects are poorly understood. Therefore, we evaluated the capacity of Pg-EE to strengthen the skin barrier and improve skin hydration. Pg-EE can enhance the expression of *filaggrin (FLG)*, *transglutaminase (TGM)-1*, *hyaluronic acid synthase (HAS)-1*, and *HAS-2* in human keratinocytes. Moreover, Pg-EE down-regulated the expression of pro-inflammatory cytokines and up-regulated the production of *FLG*, *HAS-1*, and *HAS-2* suppressed by UVB through inhibition of p38 mitogen-activated protein kinase (MAPK) and extracellular signal-regulated kinase (ERK) pathways. Given the above, since Pg-EE can improve skin barrier, hydration and reduce the UVB-induced inflammation on skin, it could therefore be a valuable natural ingredient for cosmetics or pharmaceuticals to treat skin disorders.

## 1. Introduction

Skin is the largest organ in our body and has various distinct and complex functions. It is comprised of three distinct layers: epidermis, dermis and subcutis [1]. The epidermis is the outermost layer of the skin that acts as a barrier against damage from the environment, which includes pressure, micro-organisms, and radiation [2,3,4]. Keratinocytes make up nearly 95% of the epidermis. The stratum corneum (SC) is the outermost layer of the epidermis and has a pivotal role in maintaining skin barrier integrity and skin elasticity [5]. In studies of human skin, the improvement of skin barrier function and hydration are considered important factors for maintaining skin homeostasis. Imperfections in the skin barrier can lead to several skin diseases, such as allergies, atopic dermatitis, and infections [6]. As keratinocytes are the primary cell type in the skin barrier, the renewal of the SC derived from the proliferation of keratinocytes can limit skin damage and infections caused by environmental stressors.

Hydration affects SC flexibility, which further impacts the strength and elasticity of the skin [7,8,9,10]. The level of SC hydration is a crucial factor for skin barrier function [11]. The SC controls water loss from the epidermis by preventing water evaporation from the body. SC hydration can be determined by water on the outer surface and can be improved through the use of certain pharmaceuticals and cosmetics. Such bioactive compounds can form an occluding film that separates the SC from the surrounding environment [12].

Ultraviolet (UV) radiation is one of the most destructive exogenous factors for skin health. It can cause cellular damage and clinical consequences such as sunburn, photoaging, or even skin cancer [13]. According to its wavelength, UV can be classified into UVA (400–315 nm), UVB (315–280 nm), and UVC (280–100 nm). The shorter wavelengths are associated with more severe damage. However, because UVC is almost completely absorbed by the ozone layer and atmosphere, UVB is the most damaging electromagnetic radiation. Several studies have reported the harmful effects of UVB on skin, such as skin photoaging [14,15,16], DNA damage and increased oxidative stress [17,18], and skin inflammation through various mechanisms [19,20,21,22].

Various species of *Potentilla* have long been used in traditional medicine in Asia, Europe, and North America. Some Chinese conventional medicine books record that *Potentilla glabra* var. *mandshurica* (Maxim.) Hand.-Mazz. (Pg) was prescribed against microbial infection, inflammatory diseases, and chronic metabolic diseases [23,24]. In addition, extracts from *Potentilla glabra* exhibit antioxidant, antimicrobial, anti-inflammatory, and anti-ulcerogenic properties. Previously, the anti-inflammatory effects of *Potentilla glabra* ethanol extract and its molecular mechanism have been investigated [25]. In this paper, it was reported that the extract of Pg contains three major flavonoids, quercetin, kaempferol, and naringenin, that can suppress nitric oxide (NO) production by reducing the phosphorylation of Src and ameliorate inflammatory symptoms in HCl/EtOH-induced gastritis. However, few studies have examined its functions at the cellular and molecular levels. Recently, a paper reported that Pg-EE played an anti-inflammatory role by targeting Src in NF-κB pathways, which reduced lipopolysaccharide (LPS)-induced inflammation in RAW264.7 cells as well as HCl/EtOH-induced gastritis in a mouse model [25]. In this paper, we examine the characteristics of Pg-EE in keratinocytes, which are a type of skin cell, under UVB irradiation.

## 2. Results

### 2.1. Effects of Pg-EE on Cell Viability and Skin-Protective Activities

**Figure 1:**
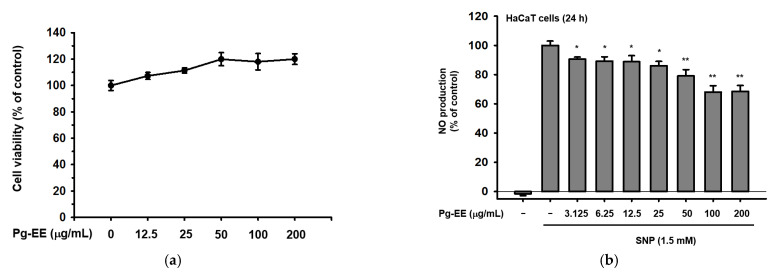

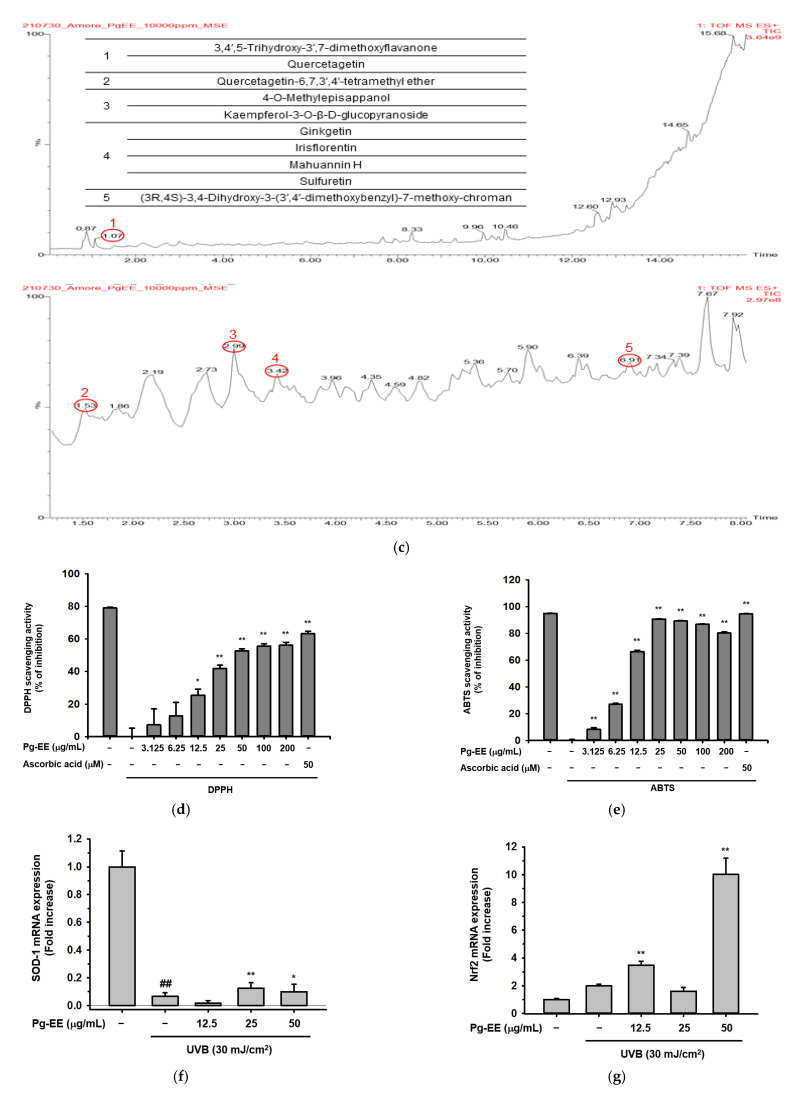

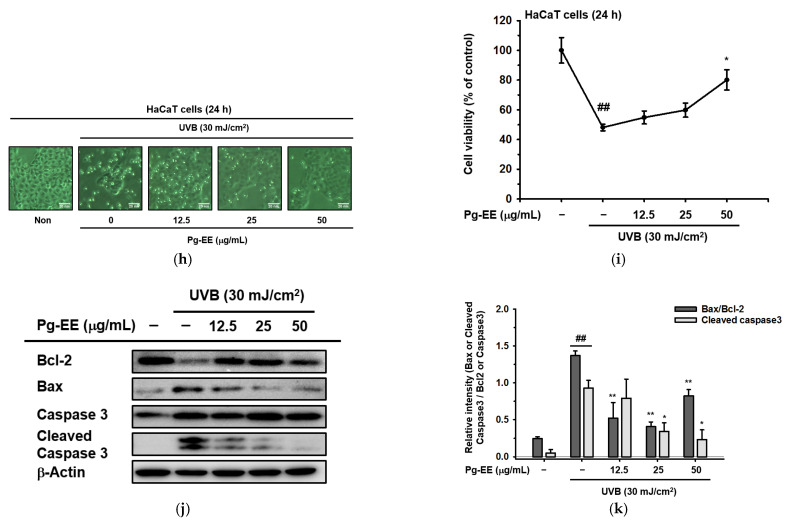
Effect of Pg-EE on cell viability and skin-protective abilities. (**a**) Cell cytotoxicity of HaCaT cells was checked by MTT assay. (**b**) NO assay was performed in HaCaT cells by coupling SNP (1.5 mM) together with Pg-EE treatments in a dose-dependent manner (3.125 to 200 μg/mL). (**c**) Ingredients of Pg-EE were analyzed by LC-MS/MS spectrometry. (**d**) Cells were incubated with Pg-EE (3.125 to 200 μg/mL) and 250 μM DPPH at 37 °C for 30 min, then absorbance was measured at 517 nm. Ascorbic acid (50 μM) together with positive controls. (**e**) ABTS and potassium persulfate solution mixed together with dose-dependent Pg-EE (3.125 to 200 μg/mL) in 37 °C incubator for 30 min. The absorbance of mixture solution was detected at 730 nm. (**f**,**g**) HaCaT cells were pretreated with Pg-EE in a dose-dependent manner (12.5, 25, and 50 μg/mL) for 30 min and irradiated with UVB (30 mJ/cm^2^). After incubating for 12 h, the mRNA levels of *SOD-1* and *Nrf2* were measured by real-time PCR. *GAPDH* was used as an internal control. (**h**) The morphology of HaCaT cells with Pg-EE treatment (12.5, 25, and 50 μg/mL) under UVB irradiation for 24 h was examined by microscopy. (**i**) Cell viability of HaCaT cells treated with Pg-EE (12.5, 25, 50, and 100 μg/mL) under UVB irradiation (30 mJ/cm^2^) for 24 h was measured by MTT assay. (**j**) Effect of Pg-EE on apoptosis of UVB-induced HaCaT cells was measured by immunoblotting analysis of active caspases. (**k**) The relative intensity of immunoblots (Bax, Bcl2, Cleaved Caspase 3, and Caspase 3) were measured by ImageJ. * *p* < 0.05 and ** *p* < 0.01 compared with control groups (SNP-treated group, only DPPH and ABTS group, only UVB group). ## *p* < 0.01 compared with normal group.

Before evaluating the characteristics of Pg-EE, we tested the viability of HaCaT cells (human keratinocytes). Because the outermost layer and middle layer of the skin are epidermis and dermis, respectively [26], it was reasoned that if Pg-EE did not harm human keratinocytes, that would be a good indication that Pg-EE should not affect cell viability or have cytotoxic effects on skin-related cells. We used MTT assays to measure the cellular metabolic activities of HaCaT cells that were treated with Pg-EE. The MTT assay is a colorimetric assay wherein metabolically active cells can reduce MTT to purple formazan crystals. In other words, the darker purple the mixture solution is, the more active cells there are [27]. The cell viability results showed that Pg-EE did not damage HaCaT cells, and even promoted cell growth slightly in a dose-dependent manner from 0 μg/mL to 200 μg/mL (Figure 1a). The phytochemical components of Pg-EE were checked by LC-MS/MS (Figure 1c). Ten compositions were identified from the extract: (1) 3,4′,5-trihydroxy-3′,7-dimethoxyflavanone and quercetagetin (RT: 1.07 min), (2) quercetagetin-6,7,3’,4’-tetramethyl ether (RT: 1.53 min), (3) 4-O-methylepisappanol and kaempferol-3-O-β-D-glucopyranoside (RT: 2.99 min), (4) ginkgetin, irisflorentin, mahuannin H, and sulfuretin (RT: 3.42 min), and (5) (3R,4S)-3,4-dihydroxy-3-(3′,4′-dimethoxybenzyl)-7-methoxy-chroman (RT: 6.91 min), in addition to three major flavonoids, quercetin, kaempferol and naringenin, as previously reported [25].

Next, a nitric oxide (NO) donor, SNP [28,29], was added into HaCaT cells to induce NO production. After 24 h, Pg-EE slightly reduced the NO production and significantly decreased the NO at high concentrations (Figure 1c). Next, anti-oxidation activity of Pg-EE was examined using DPPH and ABTS assays. DPPH assays are used to evaluate the free radical scavenging ability of natural constituents [30]. Cells were treated with Pg-EE in a dose-dependent manner from 0 μg/mL to 200 μg/mL, and the data suggested a remarkable scavenging activity even at a low dose (Figure 1b). Another antioxidant test, the ABTS assay [31], also demonstrated clear effects of Pg-EE on free radical scavenging (Figure 1d). Superoxide dismutase 1 (SOD-1) is a free radical scavenging enzyme that can act against oxygen radical species in many kinds of circumstances. Nuclear factor erythroid 2-related factor 2 (Nrf2) is an important regulator for antioxidant and cellular protective. After the HaCaT cells were irradiated by UVB, Pg-EE was able to slightly recover the mRNA level of *SOD-1* at 25 and 50 μg/mL (Figure 1e). Moreover, there was a significant enhancement of *Nrf2* at 50 μg/mL (Figure 1f), which showed that Pg-EE has good characteristics against oxidation induced by UVB irradiation. Then, to evaluate the protective effects of Pg-EE against photodamage, the morphology of HaCaT cells treated with Pg-EE was examined after irradiation with UVB at 30 mJ/cm^2^. Microscopy revealed that HaCaT cells that were only irradiated by UVB exhibited a round shape and floated on the media, which comprised the dead cells induced by UVB, compared with normal cells. However, treatment with Pg-EE reduced the occurrence of these floating round-shaped dead cells (Figure 1g). Additionally, MTT cell viability assays demonstrated that Pg-EE effectively reduced UVB-induced death of HaCaT cells (Figure 1h), which corresponded to a previous observation. To determine how Pg-EE could protect the HaCaT cells from UVB irradiation, cell death-related mechanisms were studied. Interestingly, Pg-EE relieved the UVB irradiation-induced cell death through anti-apoptosis (Figure 1i). It is well known that UVB can induce DNA damage and inflammation in cells [32]. It has also been shown that UVB irradiation can lead to apoptosis in keratinocytes [33]. B-cell lymphoma 2 (Bcl-2) and Bcl-2-like protein 4 (Bax) are the major players of the Bcl-2 family that participate in regulating apoptosis [34,35]. Bax has pro-apoptotic roles, whereas Bcl*-2* can inhibit the activity of Bax to prevent apoptosis [36,37,38]. Thus, the ratio of Bax and Bcl*-2* can act as a marker of apoptosis susceptibility in keratinocytes damaged by UVB. Here, immunoblots showed that Pg-EE suppressed Bax expression in HaCaT cells exposed to UVB but recovered the Bcl*-2* expression which reduced by UVB, suggesting that Pg-EE can help HaCaT cells resist UVB damage. In addition, caspase 3, which is a critical regulator and promoter of apoptosis [39], was cleaved during UVB-induced apoptosis. Meanwhile, Pg-EE relieved the cleavage of caspase 3 in a dose-dependent manner. The relative intensity of immunoblots was also measured (Figure 1j). Taken together, Pg-EE exhibited beneficial effects on decreasing NO production and an excellent anti-oxidative ability for free radical scavenging. Moreover, Pg-EE can prevent photodamaging and rescue keratinocytes from UVB irradiation through anti-apoptotic pathways.

### 2.2. Anti-Inflammatory Abilities of Pg-EE in Keratinocytes under UVB Irradiation

**Figure 2:**
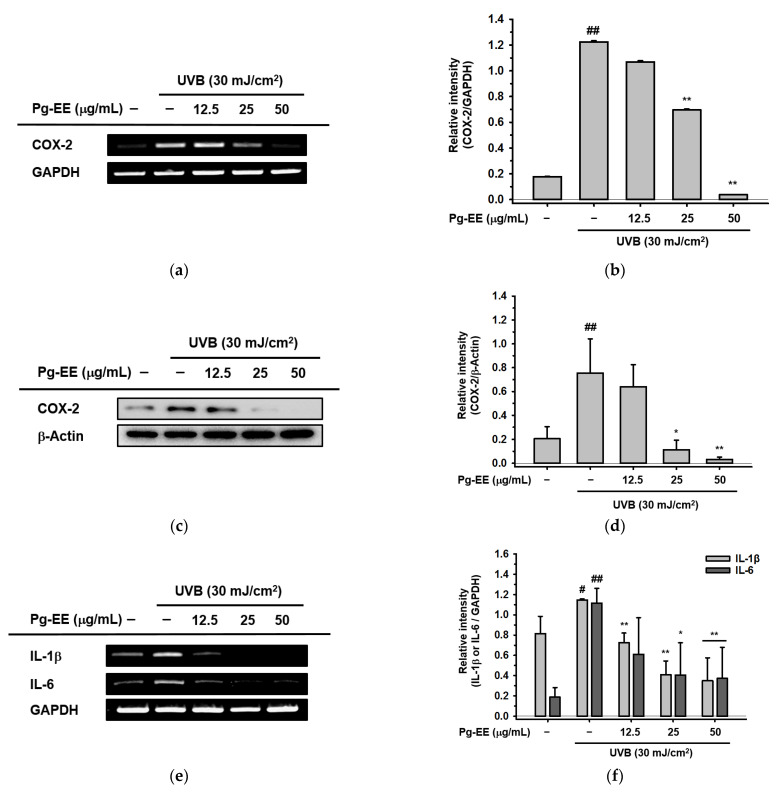
Anti-inflammatory abilities of Pg-EE in keratinocytes under UVB irradiation. (**a**) HaCaT cells were pretreated with Pg-EE in a dose-dependent manner (12.5, 25, and 50 μg/mL) for 30 min and irradiated with UVB (30 mJ/cm^2^). The cell plates were then incubated for 6 h. The mRNA level of *COX-2* was measured by RT-PCR. (**c**) HaCaT cells were irradiated by UVB (30 mJ/cm^2^) after pre-treatment with Pg-EE. Cells were then additionally incubated for 12 h. The protein level of *COX-2* in whole lysates was checked by immunoblotting analysis. (**d**) The relative intensity of immunoblot (*COX-2*) was measured by ImageJ. (**e**) HaCaT cells were pretreated with Pg-EE (12.5, 25, and 50 μg/mL) for 30 min, irradiated with UVB (30 mJ/cm^2^), then incubated for 6 h in 37 °C incubator. The mRNA levels of *IL-1β* and *IL-6* were measured by RT-PCR. (**b**,**f**) The relative intensity of RT-PCR results (*COX-2*, *IL-1β*, and *IL-6*) was measured by ImageJ. * *p* < 0.05 and ** *p* < 0.01 compared with control groups (only UVB group). # *p* < 0.05 and ## *p* < 0.01 compared with normal groups.

There are many papers reporting that UVB can elicit severe inflammatory responses leading to problems in skin [17,19,40,41,42]. UVB activates key enzymes like *cyclooxygenase-2 (COX-2)* and induces the production of many pro-inflammatory cytokines, such as *interleukin-1β (IL-1β)*, *interleukin 6 (IL-6)*, and *tumor necrosis factor alpha (TNF-α)*. *COX-2* is an inflammation-associated enzyme that is triggered by these pro-inflammatory cytokines and mediators [43], which induces the inflammatory response. These inflammatory responses cause skin damage that results in skin aging [44,45]. Therefore, to test the effects of Pg-EE on anti-inflammatory abilities affected by photodamage, the mRNA and protein expression levels of *COX-2* were measured in HaCaT cells pre-treated with Pg-EE before exposure to UVB irradiation. After treatment with Pg-EE, the UVB-induced production of *COX-2* was significantly reduced in a dose-dependent manner (Figure 2a,c). The relative intensity of *COX-2* in mRNA and protein levels were measured (Figure 2b,d). Moreover, the mRNA levels of pro-inflammatory cytokines, such as *IL-1β* and *IL-6*, which are induced by photodamage, were also down-regulated by Pg-EE in a dose-dependent manner (Figure 2e). The relative intensity of *IL-1β* and *IL-6* in mRNA levels were also checked (Figure 2f). Taken together, the high expression levels of pro-inflammatory enzyme and cytokines induced by UVB irradiation can be relieved by Pg-EE, indicating that Pg-EE has anti-photodamage activity.

### 2.3. Effect of Pg-EE on Skin Moisture Protection Activity

**Figure 3:**
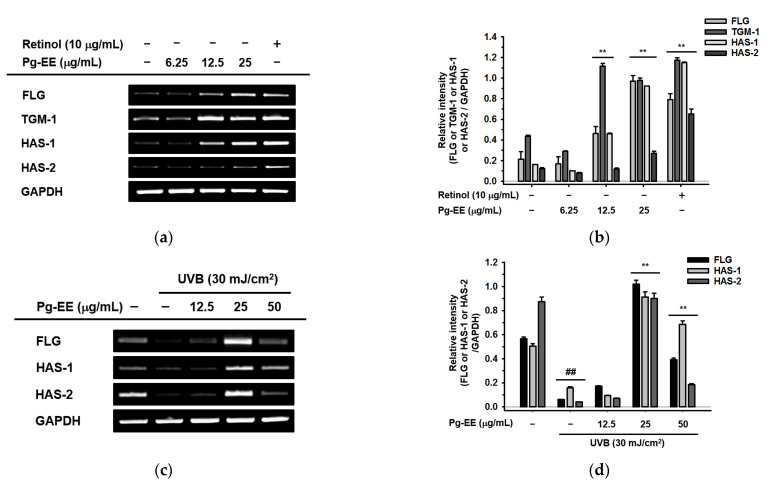

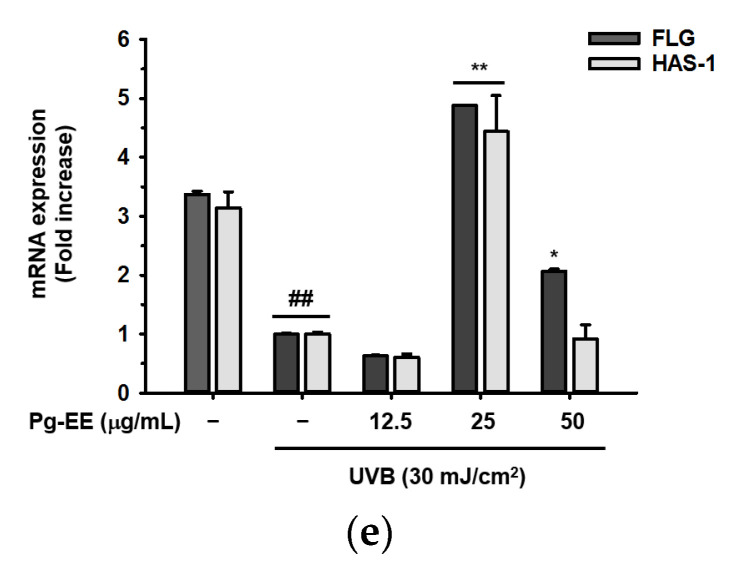
Effect of Pg-EE on skin moisture protective activity. (**a**) HaCaT cells were treated with Pg-EE (6.25, 12.5, and 25 μg/mL) or retinol (10 μg/mL) for 6 h. The mRNA levels of *FLG, TGM-1, HAS-1,* and *HAS-2* were measured by RT-PCR. (**c**) HaCaT cells were pretreated with Pg-EE (12.5, 25, and 50 μg/mL) for 30 min and irradiated with UVB (30 mJ/cm^2^). After incubation for 6 h, the mRNA levels of *FLG, HAS-1,* and *HAS-2* were measured by RT-PCR. (**e**) HaCaT cells were pretreated with Pg-EE (12.5, 25, and 50 μg/mL) for 30 min and irradiated with UVB (30 mJ/cm^2^). After incubating for 6 h, the mRNA levels of *FLG* and *HAS-1* were measured by real-time PCR. *GAPDH* was used as an internal control. (**b**,**d**) The relative intensity of mRNA levels was measured using ImageJ. * *p* < 0.05 and ** *p* < 0.01 compared with only UVB-irradiated groups. ## *p* < 0.01 compared with normal groups.

As mentioned earlier, skin barrier and hydration are crucial for skin health. Thus, enhancing moisturizing factors has been the focus of many studies. There are many moisturizing factors, such as *FLG*, *TGM-1*, and *HAS* family members. Among these factors, *FLG* is an important epidermal protein that has an essential role in maintaining skin structure and function. It also has a pivotal impact on maintaining skin homeostasis [46]. In addition, *TGM-1* is a membrane-associated gene that is highly expressed in the epidermis [47]. *TGM-1* is involved in assembly of the cornified cell envelope, which has a protective role in forming the skin barrier. In particular, *TGM-1* can synthesize strong bonds named cross-links among the structure proteins. These steady connections form the cornified cell envelope to make the epidermis stable [48,49]. Beside the skin barrier factors mentioned above, skin hydration factors such as *HAS-1* and *HAS-2* are also critical for skin health. *HAS-1* and *HAS-2* are isoenzymes that are capable of cellular hyaluronan production [50]. Most previous research has focused on *HAS-2*, whereas *HAS-1* has received less attention. However, some recent papers have suggested that *HAS-1* is related to human keratinocyte differentiation and is also correlated with the expression of HA, which indicates that *HAS-1* has a crucial role in regulating skin homeostasis [51]. To investigate whether Pg-EE played a role in protecting moisture levels in human keratinocytes, Pg-EE was added to HaCaT cells in the presence or absence of UVB irradiation. The gene expression levels of skin barrier and hydration factors were measured by RT-PCR. Pg-EE enhanced the expression of *FLG, TGM-1, HAS-1*, and *HAS-2* in a dose-dependent manner (6.25, 12.5, and 25 μg/mL) in the absence of UVB exposure (Figure 3a), whereas the expression of these moisturizing factors was inhibited at 50 μg/mL or higher doses (data not shown). Moreover, in the presence of UVB, the expression levels of *FLG, HAS-1*, and *HAS-2* were significantly reduced compared to the normal condition. However, these expression levels recovered remarkably after treatment with 25 μg/mL Pg-EE (Figure 3c). The expression levels of these three moisturizing factors slightly increased with 50 μg/mL Pg-EE but to a lesser extent than seen with 25 μg/mL Pg-EE, possibly due to inhibitory effects of the higher dose (data not shown). The expression of *TGM-1* also did not obviously recover from the exposure to UVB irradiation (data not shown). These data were confirmed by real-time quantitative PCR (Figure 3e). The relative intensity of corresponding mRNA levels conducted by RT-PCR was also measured (Figure 3b,d).

### 2.4. Pg-EE Shows Anti-Inflammatory and Moisture Protective Abilities via AP-1 Pathway under UVB

**Figure 4:**
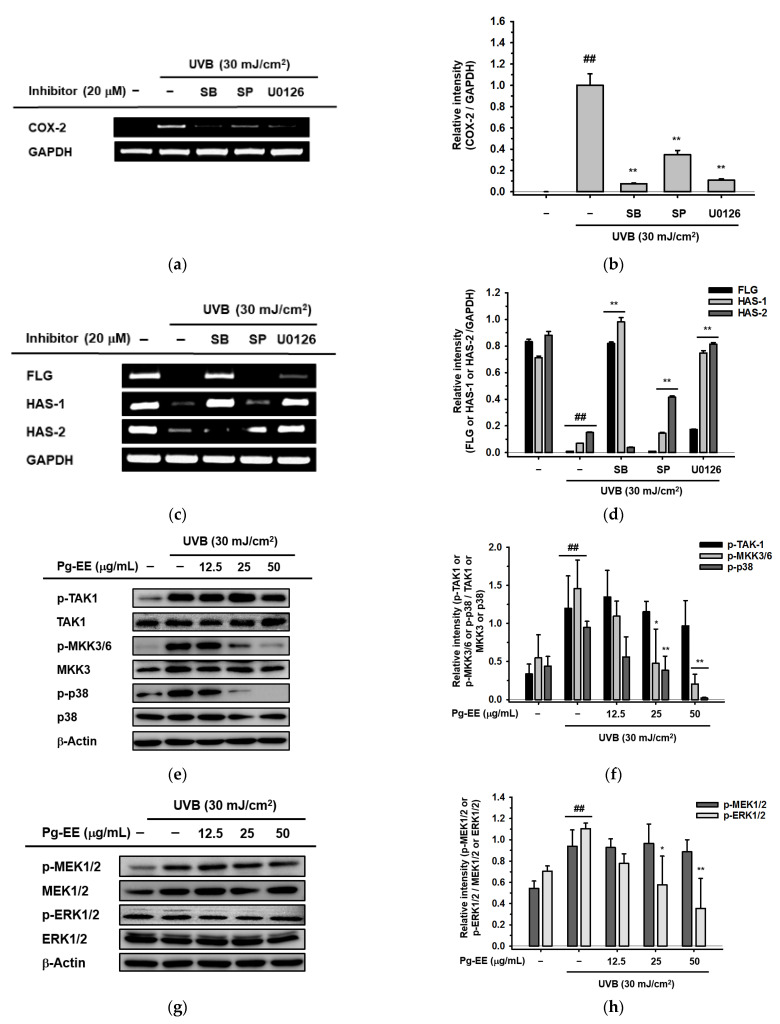

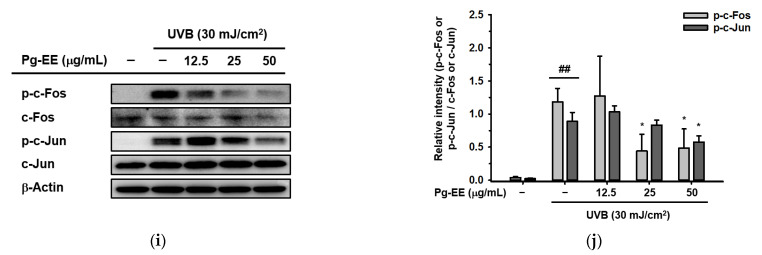
Pg-EE shows anti-inflammatory and moisture-protective abilities via AP-1 pathway under UVB irradiation. (**a**,**c**) HaCaT cells were pre-treated with MAPK inhibitors [SB203580 (a p38 inhibitor), SP600125 (a JNK inhibitor), and U0126 (an ERK inhibitor)] for 30 min. Then, plates were irradiated with UVB (30 mJ/cm^2^). After 6 h, the mRNA levels of *COX-2, FLG, HAS-1*, and *HAS-2* were measured by RT-PCR. (**e**,**g**) HaCaT cells were pre-treated with Pg-EE in a dose-dependent manner (12.5, 25, and 50 μg/mL) for 30 min and irradiated with UVB (30 mJ/cm^2^). After incubating 12 h, the protein levels of both phosphorylated and total forms of TAK1, MKK3*/6,* MEK1*/2,* p38, ERK1/2, *c-*Jun, and *c-*Fos were detected by immunoblotting analysis. (**b**,**d**,**f**,**h**–**j**) The relative intensity of mRNA (*COX-2*, *FLG*, *HAS-1*, and *HAS-2*) and protein levels (p-TAK1, p-MKK3/6, p-p38, p-MEK1/2, p-ERK1/2, p-c-Jun, and p-c-Fos) were measured by ImageJ. * *p* < 0.05 and ** *p* < 0.01 compared with control group (only UVB group). ## *p* < 0.01 compared with normal group.

Previously, studies have reported that UVB irradiation can induce the expression of inflammatory genes and suppress the production of moisturizing factor genes [52,53]. Thus, we examined whether Pg-EE displayed anti-inflammatory and moisture protective roles in human keratinocytes. We treated cells with AP-1 pathway-related inhibitors—SB203580 (a p38 inhibitor) at a dose of 20 μM, SP600125 (a JNK inhibitor) at 20 μM, and U0126 (an ERK inhibitor) at 10 μM—together with UVB irradiation. All three inhibitors reduced UVB-induced production of *COX-2* (Figure 4a). Among the three inhibitors, SB203580 and U0126 exhibited stronger decreases than SP600125. Moreover, when we examined the moisturizing effects in UVB-irradiated keratinocytes, both SB203580 and U0126 prominently rescued the expression of *FLG* and *HAS-1*, which were suppressed by UVB irradiation before (Figure 4c). In addition, SP600125 and U0126 increased the expression of *HAS-2*. Together, these data suggest that p38 and ERK signaling pathways play dominant roles in anti-inflammatory and moisture-protective abilities. Next, we investigated signaling pathways upstream of p38 and ERK. In the p38 signaling pathway, UVB activated the phosphorylation of TAK1, mitogen-activated protein kinase (MKK) 3/6, and p38. The phosphorylation of MKK3/6 and p38 showed a dose-dependent decrease by treatment with Pg-EE. However, there was no big change in p-TAK1, indicating that TAK1 was the target of Pg-EE (Figure 4e). In addition, the ERK pathway was also checked by adding Pg-EE prior to UVB-irradiated HaCaT cells. UVB activated the phosphorylation of MAPK and ERK kinases (MEK)1/2 and ERK1/2, whereas phospho-ERK1 was reduced by Pg-EE, which demonstrated that Pg-EE targeted MEK1*/2* to regulate the ERK signaling pathway (Figure 4g). In addition, c-Jun and c-Fos derived from the AP-1 signaling pathway were also suppressed by Pg-EE (Figure 4i). The relative intensity of mRNA levels determined by RT-PCR were measured (Figure 4b,d), and the relative intensity of immunoblots (p-TAK1, p-MKK3/6, p-p38, p-MEK1/2, p-ERK1/2, p-c-Jun, and p-c-Fos) was also tested, respectively (Figure 4f,h,j). Taken together, Pg-EE down-regulated the expression of inflammatory genes and up-regulated the production of skin barrier and hydration genes through p38/ERK/AP-1 signaling pathways under UVB irradiation.

## 3. Discussion

Skin health is an important topic for clinical researchers. Among the focuses of such studies, moisturizing ability is one of the basic characteristics of cosmetic applications. As previously mentioned, UVB irradiation can cause a loss of skin moisture and induce inflammation. These bad influences can finally lead to the extrinsic skin aging called photoaging. Photoaging is a very complex change for human skin. There are two main factors that influence this process; one is skin type, and the other is ethnicity. First, photoaging more prevalently occurs in people whose skin is fair. Because people who have darker skin have more melanin content, which shows a more powerful ability to protect the skin from photo damage [54,55]. Second, ethnicity is also a predominant factor affecting pigmentary changes and the degree of wrinkles between white- and yellow-skinned people [56]. UVB is one of the primary ways that photoaging is induced. The cellular DNA located in the epidermis can absorb UVB irradiation, which causes skin damage, such as sunburn [56]. In addition, UV irradiation generates reactive oxygen species (ROS). ROS lead to oxidative damage in cell membranes or DNA. In clinical trials, photoaging causes a loss of translucency, laxity, wrinkles, inflammation, and so on [57]. Because of these, many treatments for photoaging have been developed by scientists in both topical and procedural interventions. The classical methods include topical retinoids, cosmeceuticals, injectable soft-tissue fillers, etc. [55].

Retinoids are a group of natural or synthetic compounds related to vitamin A, which can treat the mild or moderate photoaging. First, retinoids are pivotal for the differentiation of keratinocytes, the regeneration of the epidermis, and increasing the density of SC [58], which forms a thicker barrier to protect against UV irradiation. Second, retinoids enhance the production of collagen and improve elastic fiber, relieving not only wrinkles, but also the laxity of the skin [59,60]. Third, retinoids are beneficial in reducing the inflammation induced by photodamage by eliminating the release of pro-inflammatory cytokines [61]. 5-fluorouracil cream is also used for improving photoaging by increasing type 1 procollagen and activating dermal remodeling [62]. It can also accelerate the wound healing of injured epidermis.

Because of the ROS produced by UV irradiation, some antioxidants have also been proved to have anti-photoaging effects via scavenging the free radicals. Therefore, vitamin C and E, coenzyme Q, and lipoic acid have been confirmed to have high abilities in preventing oxidation and photoaging [62]. Besides the single compounds, some natural extracts such as ginseng extracts also have antioxidant, anti-photoaging and anti-inflammatory abilities. A number of papers show that Korean red ginseng can relieve photoaging, increase the synthesis of collagen, and reduce skin inflammation [63]. Currently, people are paying great attention on their own skin health and there is increasing focus on natural products. Thus, finding a new herbal ingredient that simultaneously possesxes many different anti-photoaging properties in skin is an interesting area to study for researchers. The effects of keratinocytes irradiated with UVB have been reported by many papers. Cui et al. found that the ratio of Bcl-2 and Bax decreased, while p-ERK1/2 was up-regulated by UVB [64]. Chiu et al. demonstrated that the p38 signaling pathway was also involved in UVB-induced photoaging in keratinocytes [65]. Ko et al. also found that Nrf2 was suppressed by UVB and alleviated by ergothioneine [66]. All of these results were confirmed by our data in some ways. Pg is an attractive herb that mostly grows in mountainous areas. Therefore, in the past, people living near the mountains used Pg to treat colds, stomachaches, and especially irregular periods in women.

**Figure 5:**
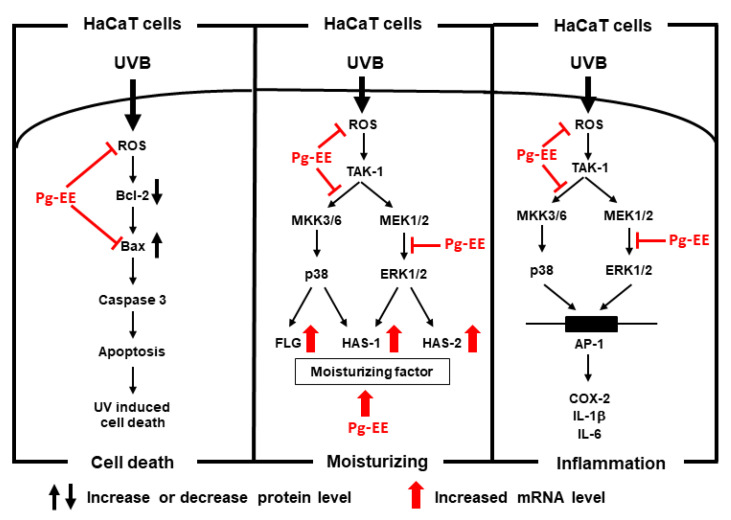
The schematic pathway of Pg-EE on skin protection against the photoaging process. The schematic illustration of Pg-EE-mediated skin-protective effects on the anti-photoaging process is summarized in terms of cell death, moisturizing, and inflammation.

In our study, we found that Pg-EE not only has good antioxidant activity, but also protects skin against UVB irradiation, has good anti-inflammatory capacity, and helps skin retain moisture. Our data exhibited that Pg-EE improved skin hydrating effects by elevating moisturizing factors, which were inhibited by UVB before. Pg-EE can enhance the *FLG, TGM-1, HAS-1*, and *HAS-2* in a dose-dependent manner alone. Whereas it can remarkably recover the *FLG*, *HAS-1*, and *HAS-2* at a dose of 25 μg/mL in the presence of UVB, showing that Pg-EE can repair the loss of water of skin caused by UVB. Further study revealed that Pg-EE recovered the level of *FLG* by decreasing the phosphorylated MKK3/6/p38 pathway and gathered the expression of *HAS-2* by downregulated the phosphorylation of ERK1*/2* pathway. Both the decrease of p38 and ERK led to the re-accumulation of *HAS-1*. In addition, our study also illustrated that Pg-EE has a crucial role in skin protection because it can reduce the abnormal immune response and DNA damage caused by UVB. Pg-EE reduced the expression of *COX-2*, *IL-1β*, and *IL-6* by controlling the p38, ERK, and AP-1 pathway, while acting against apoptosis by restraining the ratio of Bax and Bcl*-2*. Because free radicals play an important role in inducing skin aging and oxidant activity can induce apoptosis, Pg-EE has both ideal antioxidant and anti-apoptotic activities, which means Pg-EE can be a potential anti-aging cosmetics ingredient. In summary, Pg-EE can enhance skin moisturizing levels and prevent keratinocytes from UVB-induced photodamage by suppressing the p38/ERK/AP-1 pathway, as well as by inhibiting apoptosis (Figure 5).

Taken together, because Pg-EE did not cause cytotoxicity, it becomes a potential remedy for use in preventing UVB-induced skin photoaging, and also has a high-efficiency moisturizing capacity. This is the first application of Pg-EE for human skin health protection. However, although Pg-EE exhibited a variety of skin-protecting properties, it still has undefined aspects that need to be solved in the future, such as stability of the Pg-EE compounds. In addition, whether Pg-EE has some other functions with respect to the skin, such as anti-melanogenesis, antioxidant, or anti-aging properties, remains to be further explored. Finally, since Pg-EE effectively prevents human keratinocytes from UVB photodamage and protects skin moisture, it is suggested that this extract could be applied in the development of a cosmeceutical product.

## 4. Materials and Methods

### 4.1. Materials

The HaCaT (human skin keratinocyte) was purchased from the CLS Cell Lines Service GmbH (Eppelheim, Germany). 1,1-Diphenyl-2 picrylhydrazyl radical (DPPH), 2,2′-azino-bis (3-ethylbenzothiazoline-6-sulfonic acid) diammonium salt (ABTS), sodium nitroprusside (SNP), (3-4,5-dimethylthiazol-2-yl)-2,5-diphenyl-tetrazolium bromide (MTT), dimethyl sulfoxide (DMSO), sodium dodecyl sulfate (SDS), and bovine serum albumin (BSA) were bought from Sigma (St. Louis, MO, USA). SB203580 (a p38 inhibitor), SP600125 (a JNK inhibitor) and U0126 (an ERK inhibitor) were also acquired from Sigma (St. Louis, MO, USA). Fetal bovine serum (FBS) was obtained from Gibco (Grand Island, NY, USA). Dulbecco’s Modified Eagle’s medium (DMEM), 0.25% Trypsin solution and the antibiotic reagents (penicillin and streptomycin) were purchased from HyClone Laboratories (Logan, Utah, USA). 1X phosphate-buffered saline (PBS) was purchased from Samchun Pure Chemical Co. (Gyeonggi-do, Korea). TRIzol reagent was purchased from Molecular Research Center, Inc. (Cincinnati, OH, USA). The sets of primers for polymerase chain reaction (PCR) were synthetized by Macrogen (Seoul, Korea), and PCR premix was obtained from Bio-D Inc. (Seoul, Korea). Total and phospho-forms of antibodies were purchased from Cell Signaling Technology (Beverly, MA, USA), and c-Fos and β-actin were bought from Santa Cruz Biotechnology, Inc. (Dallas, TX, USA). All of the antibodies were diluted 1:2500.

### 4.2. Preparation of Pg-EE and Liquid Chromatography–Tandem Mass Spectrometry (LC-MS/MS)

Pg-EE was prepared from *Potentilla glabra* var. *mandshurica* (Maxim.) Hand.-Mazz. (1 kg) by extraction with 95% ethanol in an ultrasonic extractor (Ultrasonic Cleaner UC-10, UC-20, 400 W) for 4 h at 50 °C (three times) to yield 21.2 g [25]. After removal of the solvent under reduced pressure in vacuo, the extract was freeze-dried for 48 h at −80 °C, and then stored in a freezer at −20 °C until use. The LC-MS/MS analysis was performed on a Xevo G2-XS Q-TOF-LC/ MS (Waters, USA), as reported previously [67].

### 4.3. Cell Culture

HaCaT cells were cultured in DMEM media containing 10% FBS and 1% penicillin/streptomycin antibiotics. The cells were maintained in a 5% CO_2_ incubator (Thermo Fisher Scientific, Waltham, MA, USA) at 37 °C.

### 4.4. Compound Treatment

The primary stock compound of Pg-EE was dissolved in 100% dimethyl sulfoxide (DMSO) at a concentration of 100 mg/mL. For each experiment, the primary stock was diluted with media at doses ranging from 12.5 μg/mL to 50 μg/mL for in vitro assays.

### 4.5. Cell Viability Assay

HaCaT cells were spread into 96-well plates at 1 × 10^5^ cell/mL in DMEM media. After incubating overnight, Pg-EE was added in a dose-dependent manner. After 24 h, 100 μL cultured media was removed, and 10 μL of 5 mg/mL MTT solution was injected into each well. After 3 h, MTT stopping solution was added when purple formazan appeared. After 18 h, the absorbance of each well was detected at 570 nm using a microplate reader (BioTek Instruments Inc., Winooski, VT, USA).

### 4.6. Nitric Oxide (NO) Assay

HaCaT cells were spread into 96-well plates at a density of 1 × 10^6^ cells/mL. Then, cells were treated with Pg-EE in a dose-dependent manner and incubated for 24 h. Next, 100 μL supernatant was transferred to each well of a fresh 96-well plate. Griess reagent was applied and mixed with these supernatants at the same volume (1:1). Finally, the plates at the absorbance of 540 nm were read by a multi-plate reader [68]. Then, the NO standard curve (y = ax + b) was used to calculate the NO expression of each group ‘x = (y − b)/a’. In the equation, the x axis represents NO production, and the y axis represents absorbance. Finally, the percent of NO production of each group was counted on the basis of the positive control.

### 4.7. DPPH Assay

DPPH is a method that was developed to predict antioxidant activities. The scavenging of DPPH radicals can be used to determine free radical scavenging capacity. First, primary stocks were made of DPPH (3 mM) in methanol, L-Ascorbic Acid (50 mM) in LC-MC grade water, and Pg-EE (100 mg/mL) in DMSO, respectively. Then, the DPPH stock solution was diluted with LC-MC-grade water to 250 μM and Pg-EE was diluted to 20 mg/mL. Next, serial dilutions were made of Pg-EE from 200 μg/mL to 0 μg/mL and fully mixed with 250 μM DPPH sister solution at a ratio of 1:100. These mixtures were incubated at 37 °C for 30 min before detecting the absorbance at 517 nm.

### 4.8. ABTS Radical Scavenging Assay

ABTS radical scavenging assay is another method that can evaluate antioxidant scavenging effects. Before the experiment, 7.4 mM ABTS and 2.4 mM potassium persulfate were mixed at a ratio of 1:1. Then, the mixture was wrapped with foil and incubated in a 37 °C incubator for 30 min until the solution color changed to dark green. Then, the ABTS mixture was diluted until the absorbance was between 0.72 and 0.74 at 730 nm. Next, serial dilutions of Pg-EE were made from 400 μg/mL to 0. Ascorbic Acid (50 mM) was also mixed with ABTS independently as positive control. After each of the chemicals was diluted in DPBS (Pg-EE from 400 μg/mL to 0 and Ascorbic Acid 50 μM), Pg-EE or ascorbic acid was mixed with ABTS and incubated in a 37 °C incubator for 30 min. Finally, the absorbance was detected at 730 nm.

### 4.9. UVB Irradiation

HaCaT cells were spread evenly in a 6-well plate at a density of 1 × 10^5^ cells/mL with DMEM. Cells were pre-treated with dose-dependent Pg-EE (0–50 μg/mL) for 30 min. Next, each well was washed with 1 mL PBS once and covered the cells by 1 mL PBS. The plate was exposed in a UVB lamp (Bio-Link BLX-312; Vilber Lourmat, Collégien, France) with an emission wavelength peak of 312 nm at 30 mJ/cm^2^ [69]. After that, the PBS was removed, and the cells were re-treated with Pg-EE for 24 h.

### 4.10. Cell Morphology Photography

HaCaT cells were seeded into 6-well plates at a cell density of 1 × 10^5^ cells/mL then pre-treated with Pg-EE (from 50 μg/mL to 0 μg/mL) for 30 min. Cells were then washed with PBS and irradiated under the UVB lamp. PBS was removed and the cells were re-treated with Pg-EE in a dose-dependent manner. After 24 h, photos were taken by using an epifluorescence microscope (Olympus, Tokyo, Japan).

### 4.11. Immunoblotting Analysis

HaCaT cells were seeded into 6-well plates (SPL Life Sciences Co., Gyeonggi-do, Korea) with 10^5^ cells/well and treated with Pg-EE with the dose dependency (0, 12.5, 25, or 50 μg/mL). Then, the cells were lysed with lysis buffer (50 mM pH 7.5 Tris-HCl, 20 mM NaF, 25 mM pH 7.5 β-glycerol phosphate, 120 mM NaCl, 2% NP-40, and phosphatase/protease inhibitors). Cell lysates were centrifuged at the speed of 13,000 rpm for 15 min at 4 °C to settled cell debris. Protein concentrations were quantified by Bradford protein assay (BIO-RAD, Hercules, CA, USA). Equal amounts (20 μg protein/lane) of samples were separated by Tris-glycine SDS gels and then transferred to PVDF membranes (Millipore, Billerica, MA, USA). The membranes were blotted with 3% BSA for 1 h at room temperature and were then washed with tris-buffered saline (50 mM Tris-Cl, pH 7.5, 150 mM NaCl) and 0.1% Tween-20 (TBST) three times for 10-min intervals. The membranes were then incubated with primary antibodies overnight at 4 °C. Then, after washing with TBST three times for 10 min each interval, the membranes were incubated in the secondary antibody for 2 h at room temperature [70]. After that, the membranes were washed with TBST as before. Finally, the membranes were exposed by EzWestLumi plus (ATTO Corporation, Taito-ku, Tokyo, Japan).

### 4.12. Analysis of mRNA Levels by Semi-Quantitative Reverse Transcription–Polymerase Chain Reaction (RT-PCR) and Quantitative Real-Time Polymerase Chain Reaction (Real-Time PCR)

To evaluate the gene expression of factors related to inflammation and moisturization, HaCaT cells were seeded, pre-treated with Pg-EE, and then irradiated by UVB as described above. Additionally, SB203580 (a p38 inhibitor), SP600125 (a JNK inhibitor) and U0126 (an ERK inhibitor) were used as positive controls. Total RNA was isolated using TRIzol reagent according to the manufacturer’s instructions. Two PCRBIO HS Taq PreMix (PCR Biosystems Ltd., Oxford, UK) were used for RT-PCR, with a cycle of 30 [71]. SYBR Green Premix Ex Taq (Takara Bio, Inc., Shiga, Japan) was used together with the CFX96 Touch Real-Time PCR Detection System (Bio-Rad Laboratories, Inc., Hercules, CA, USA), with the cycle of 40 [72]. The results were normalized to endogenous *GAPDH*. The results were analyzed using the 2^−ΔΔC^_T_ method. The primers for this study are listed in Table 1.

### 4.13. Statistical Analyses

All the data are presented as means ± standard deviations, and each experiment was performed as two or three replications. All the immunoblots were measured using ImageJ. Results were analyzed using the Mann–Whitney U test to compare the statistical differences. A *p*-value < 0.05 was considered statistically significant. All statistical analyses were conducted using SPSS Statistics 25.0 (IBM, Armonk, New York, NY, USA).

## 5. Conclusions

In this study, we evaluated the effects of Pg-EE on skin protection and anti-inflammation under UVB irradiation. Pg-EE can not only enhance the skin barrier and hydration through down-regulating the p38 and ERK signaling pathways, it can also relieve the damage to human keratinocytes by reducing pro-inflammatory factors. Moreover, our data showed that Pg-EE can prevent HaCaT cells from apoptosis induced by UVB irradiation by decreasing the protein levels of Bax and cleaved caspase 3, which can be used as a good ingredient in cosmetics for the prevention of photoaging.

## Figures and Tables

**Table 1:** List of primers synthesized for semi-quantitative RT-PCR and quantitative real-time PCR.

Name		Sequence (5′ to 3′)
**Semi-quantitative RT-PCR**		
*FLG*	F	AGGGAAGATCCAAGAGCCCA
	R	ACTCTGGATCCCCTACGCTT
*TGM-1*	F	GAAATGCGGCAGATGACGAC
	R	AACTCCCCAGCGTCTGATTG
*HAS-1*	F	CCACCCAGTACAGCGTCAAC
	R	CATGGTGCTTCTGTCGCTCT
*HAS-2*	F	TTCTTTATGTGACTCATCTGTCTCACCGG
	R	ATTGTTGGCTACCAGTTTATCCAAACG
*COX-2*	F	GGGATTTTGGAACGTTGTGAA
	R	CGACATTGTAAGTTGGTGGACTGT
*IL-1β*	F	ATTTGAATTCCCTGGGTGAG
	R	CCTCATCCTGGAAGGTCCAC
*IL-6*	F	TACCCCCAGGAGAAGATTCC
	R	TTTTCTGCCAGTGCCTCTTT
*GAPDH*	F	GCACCGTCAAGGCTGAGAAC
	R	ATGGTGGTGAAGACGCCAGT
**Quantitative real-time PCR**		
*FLG*	F	GGGCACTGAAAGGCAAAAAG
	R	CACCATAATCATAATCTGCACTACCA
*HAS-1*	F	CAGCCTGCGATACTGGGTAG
	R	GCCGGTCATCCCCAAAAGTA
*SOD-1*	F	AAGCGGTGAACCAGTTGTGT
	R	GCCAATGATGGAATGCTCTC
*Nrf-2*	F	ACATCCTTTGGAGGCAAGAC
	R	TCGGGTCATTGTGAGTCAGT
*GAPDH*	F	CACTCACGGCAAATTCAACGGC
	R	GACTCCACGACATACTCAGCA

F: forward primer, R: reverse Primer.

## Data Availability

The data used to support the findings of this study are available from the corresponding author upon request.

## Sample Availability

Sample of the compound, Pg-EE is available from the authors.

## Abbreviations

FLG	Filaggrin
TGM-1	Transglutaminase-1
HAS	Hyaluronic acid synthase
MAPKs	Mitogen-activated protein kinases
ERK	Extracellular signal-regulated kinase
SC	Stratum corneum
LPS	Lipopolysaccharides
COX-2	Cyclooxygenase-2
IL-1β	Interleukin-1β
IL-6	Interleukin 6
TNF-α	Tumor necrosis factor alpha
MKK	Mitogen-activated protein kinase
MEK	MAPK or ERK kinases
